# Differential metabolic pathways underlie THC- and CBD-mediated inhibition of B-cell activation in both young and aged mice

**DOI:** 10.3389/fimmu.2025.1605474

**Published:** 2025-06-17

**Authors:** Zhanna Yekhtin, Dmytro Petukhov, Iman Khuja, Natalya M. Kogan, Reuven Or, Osnat Almogi-Hazan

**Affiliations:** ^1^ Laboratory of Immunotherapy and Bone Marrow Transplantation, Hadassah Medical Center, The Faculty of Medicine, Hebrew University of Jerusalem, Jerusalem, Israel; ^2^ Department of Molecular Biology, Institute of Personalized and Translational Medicine, Ariel University, Ariel, Israel

**Keywords:** B lymphocytes, cannabinoid, Δ^9^-tetrahydrocannabinol (THC), cannabidiol (CBD), immune activation, aged, metabolome, PDL 1

## Abstract

**Objective:**

B lymphocytes play a crucial role in immunity but also contribute to the pathogenesis of various diseases. Cannabis plants produce numerous biologically active compounds, including cannabinoids. The two most studied phytocannabinoids are Δ9-tetrahydrocannabinol (THC) and cannabidiol (CBD). These cannabinoids exert diverse and potent biological effects primarily through the endocannabinoid system (ECS), which also plays a key role in mature B-cell function. Both the immune system and the ECS undergo age-related changes that lead to a clinically significant decline in function.

**Methods:**

This study compares the effects of THC and CBD on B-cell activity in young and aged mice. Murine B lymphocytes were activated using lipopolysaccharide (LPS) and interleukin-4 (IL-4), and the impact of cannabinoid treatments was assessed in terms of cell phenotype, proliferation, antibody secretion, tumor necrosis factor-alpha (TNFα) secretion, extracellular signal-regulated kinase (ERK) phosphorylation, and the cellular metabolome.

**Results:**

Both THC and CBD exhibited dose-dependent inhibitory effects on B-cell activation in young and aged mice. However, we show here, for the first time, that the treatments induce distinct metabolic profiles. Although some metabolites, such as glucose-6-phosphate, pentose phosphate pathway (PPP) and nucleotide metabolites, were reduced by both cannabinoids, THC selectively reduced the levels of a distinct set of amino acids, while only CBD increased the levels of Citrulline and Allantoin. Additionally, the effects of THC and CBD differed between young and aged B cells, suggesting that age-related changes in the ECS may influence cannabinoid sensitivity.

**Conclusions:**

These findings provide insights into the distinct mechanisms by which THC and CBD regulate immune activation and may open the door for investigating the mechanisms behind cannabinoids effects on the immune system. They also highlight the need for further research into phytocannabinoid-based therapies, particularly in age-specific contexts. Given the immunoregulatory properties of cannabinoids, especially CBD, tailored therapeutic strategies may enhance their clinical applications

## 1 Introduction

The medical use of cannabis and its derivatives has been legalized in recent years by many countries worldwide. Cannabinoid-based treatments are now employed for specific indications, such as chronic pain, cancer palliative care, posttraumatic stress disorder, and neurological diseases. However, the lack of experimental data remains a significant barrier to the broader use of medicines containing cannabinoid active substances. Therefore, it is crucial to investigate the various biological effects of medical cannabis and its active compounds.

Cannabis plants (genus Cannabis) produce a variety of physiologically active compounds, including over 60 molecules classified as cannabinoids (1). The two most extensively studied phyto-cannabinoids (natural plant cannabinoids) are Δ^9^-tetrahydrocannabinol (THC) and cannabidiol (CBD). These cannabinoids have distinct effects on the nervous system (2): THC is a psychoactive molecule, while CBD is considered neuroprotective. However, many studies have demonstrated similar anti-inflammatory effects for both cannabinoids (3).

The diverse and powerful effects of phyto-cannabinoids on the human body are mediated by endogenous cannabinoid receptors and are affected by the levels of their endogenous ligands. The endocannabinoid system (ECS) is a central regulatory system involved in various physiological processes, including a key role in immune regulation (3).

B lymphocytes are central players in the adaptive immune response. These cells are essential for detecting danger signals and presenting antigens to T cells. Upon activation, B cells acquire effector and regulatory functions, leading to antibody production, cytokine secretion, and the development of humoral immune responses that neutralize antigens. B cells also participate in the regulation of cellular immune responses. Specific metabolic pathways are activated in B lymphocytes to meet the energetic demands associated with their maturation status and functional orientation (e.g., tolerance, effector, or regulatory activities) (4–7).

Our research, along with studies from other groups, has highlighted the role of the ECS in regulating immune function (3, 8, 9). For instance, cannabinoid receptor 2 (CB2) is involved in positioning and retaining B cells within the splenic marginal zone (10) and can regulate vaccine-induced immunity (11). CB2 has also been identified as an important factor in cancer response regulation (12).

B cells undergo significant changes with age, both in mice and humans (13). Aging decreases B cell differentiation in the bone marrow and reduces the output of mature B cells. Age-related changes include a redistribution of B cell subsets in the periphery, resulting in a marked increase in pro-inflammatory B cells (14, 15). This shift contributes to a higher frequency and severity of infectious diseases and reduced vaccine efficacy. ECS function is altered by age-associated changes as well (16, 17). In a previous study, we demonstrated a decrease in cannabinoid receptor expression on peritoneal macrophages in aged mice (9).

This study aims to compare the effects of THC and CBD treatments on the activity of B cells from young and elderly mice. Our results demonstrate that both THC and CBD treatments exert dose-dependent inhibitory effects on B cell activation in cells from both young and old mice. However, these treatments induce distinct metabolic profiles in the cells. These findings may provide insights into the different mechanisms by which THC and CBD regulate immune activation.

Understanding the reciprocal relationship between cannabinoids and immune function in both young and aged populations is essential for developing therapeutic strategies that will facilitate the clinical integration of cannabinoids.

## 2 Materials and methods

### 2.1 Mice

Two-month-old and eight-month-old C57BL/6 female and male mice were purchased from Envigo (Jerusalem, Israel). Young mice were acclimated for at least three days before the experiment in the specific pathogen-free facility of the Authority for Biological and Biomedical Models at the Hebrew University of Jerusalem, under Association for Assessment and Accreditation of Laboratory Animal Care (AAALAC) International accreditation. Aged mice were maintained in the SPF facility until they reached 18 months of age. The study was approved by the Institutional Animal Care and Use Committee of the Hebrew University of Jerusalem, in accordance with national laws and regulations for the protection of animals (MD-20-16413-1).

### 2.2 Reagents

This research was performed under the approval of The Medical Cannabis Unit in the Israeli Ministry of Health (REQ46). CBD was purchased from STI Pharmaceuticals Ltd., Newtown, UK. Δ^9^-THC was prepared in the Kogan lab at Ariel University: 3 A total of 3.14 g of CBD was dissolved in 100 mL of dry dichloromethane (DCM), and 400 mg of pre-heated MgSO_4_ was dispersed in the solution. Then, 25 μL of BF3 diethyl etherate was added, and the reaction was stirred at -15°C for 1.5 hours under an N_2_ atmosphere. The reaction was quenched with 20 mL of ice-cold saturated NaHCO_3_ solution, and the phases were separated. The aqueous phase was extracted twice with 30 mL portions of DCM. The combined organic phase was washed to neutrality with saturated NaCl solution, dried over MgSO_4_, and evaporated. The yield of Δ^9^-THC was 82.3%, with the remaining material being unreacted CBD. The purity of the THC used for treatment was >97%, as confirmed by liquid chromatography–mass spectrometry (LC-MS). Cell treatment concentrations were chosen based on viability assays (see **Supplementary Figure S2**) and on our previous experience with murine splenocytes and peritoneal macrophages (8, 9).

Antibodies for immunoblotting included anti-p-ERK1/2 (Thr202/Tyr204) and anti-ERK1/2 from Cell Signaling Technology (Danvers, MA, USA) and anti-GAPDH from Abcam (Boston, MA, USA). Secondary goat anti-rabbit HRP was obtained from Jackson ImmunoResearch Laboratories (West Grove, PA, USA). Flow cytometry antibodies, including anti-B220, anti-CD69, anti-PD1 and anti-PDL1, were obtained from BioLegend (San Diego, CA, USA). All antibodies are listed in **Table 1**.

**Table 1:** List of antibodies.

Antibody	Fluorophore	Clone
B220	APC	RA3-6B2
PDL1	PE	10F.9G2
CD69	Pacific Blue	H1.2F3
PD-1	PE	29F.1A12
CD3	FITC	145-2C11
CD11b	APC-Cy7	M1/70
p-ERK1/2	-	D13.14.4E
ERK1/2	-	137F5
GAPDH	-	EPR16891

### 2.3 B lymphocyte activation assays

#### 2.3.1 Surface marker expression analysis

Spleens were harvested from healthy C57BL/6 mice. Splenocytes were centrifuged using a Lymphoprep gradient (Serumwerk Bernburg AG, Bernburg, Germany), and mononuclear cells were isolated from the interphase layer. A total of 1 × 10^6^ splenocytes/well were plated in 24-well flat-bottom plates with RPMI 1640 medium supplemented with 10% fetal calf serum (FCS), 1% penicillin/streptomycin, and 1% L-glutamine (Sartorius, Beit Haemek, Israel). Cells were activated for 24 hours with 10 μg/mL lipopolysaccharide (LPS) and 20 ng/mL recombinant murine IL-4 (PeproTech, Rocky Hill, NJ) in the presence of the indicated cannabinoid concentrations. Next, non-adherent cells were collected and washed with cold FACS Buffer (PBS, 4% FBS). Cells were stained by fluorescent antibodies for B220, CD69, PDL1 and the appropriate isotype controls in the recommended dilutions for 30 minutes in 4°C. The cells were then washed again and pellets were resuspended in FACS Buffer. Flow cytometry analysis was done using MACSQuant analyzer. Results were finally analyzed using Fcs express 6 by *De Novo* Software. Expression levels, relative to activated non-treated cells, were calculated from the geometric mean (GM) values.

#### 2.3.2 Proliferation analysis

Splenocytes were washed and labeled with the CellTrace CFSE proliferation kit (Invitrogen, Oregon, USA). A total of 4 × 10^5^ labeled cells/well were plated in 96-well flat-bottom plates with RPMI 1640 medium supplemented with 10% FCS, 1% penicillin/streptomycin, and 1% L-glutamine. Cells were activated for four days with 10 μg/mL LPS and 20 ng/mL IL-4 in the presence of the indicated cannabinoid concentrations. Cells were then washed in PBS and stained with anti-B220 antibodies. CFSE levels in B cells were analyzed by flow cytometry.

#### 2.3.3 TNFα secretion assay

B cells were isolated by negative selection using the EasySep Mouse Pan-B Cell Isolation Kit (STEMCELL Technologies, Canada), following the manufacturer’s protocol. A total of 1 × 10^6^ cells/well were plated in 24-well flat-bottom plates and activated for 24 hours with LPS and IL-4 in the presence of the indicated cannabinoid concentrations. TNFα concentrations in the supernatant were measured using the ELISA MAX™ Deluxe Set Mouse TNFα, Cat No. 430904 (BioLegend), following the manufacturer’s protocol.

#### 2.3.4 IgG secretion assay

A total of 1 × 10^6^ splenocytes/well were plated in 24-well flat-bottom plates with RPMI 1640 medium supplemented with 10% FCS, 1% penicillin/streptomycin, and 1% L-glutamine. Cells were activated with LPS and IL-4 in the presence of the indicated cannabinoid concentrations for 72 hours. Culture supernatants were collected and stored at -80°C until use. IgG levels were measured using an ELISA assay (Cat No. 501240, Cayman Chemical, MI, USA), following the manufacturer’s protocol.

#### 2.3.5 Western blot analysis

Isolated B cells were pre-incubated for four hours at 37°C with IL-4, followed by activation with LPS for 30 minutes. Cannabinoid treatments were added 15 minutes before activation. ERK phosphorylation levels were assessed using western blot analysis. Cells were washed with PBS, and proteins were extracted from B cell pellets using RIPA lysis buffer (Bioprep, Israel) containing a protease inhibitor cocktail and phosphatase inhibitor cocktail III (Bioprep, Israel). Protein samples were subjected to reducing SDS-PAGE, followed by immunoblotting with anti-ERK and anti-p-ERK antibodies (Cell Signaling Technology). Immunoreactive proteins were detected using ECL (Bio-Rad, USA). GAPDH immunostaining was used as a loading control for normalization.

#### 2.3.6 Metabolomics analysis

Isolated B cells from young (n = 4) and aged mice (n = 3) were activated for 24 hours in the presence of 5 µg/mL THC or CBD, washed, and stored at -80°C. Frozen pellets were analyzed using LC-MS metabolomics at the interdepartmental unit of Hadassah-Hebrew University Medical School. Data processing and analysis were performed using Compound Discoverer 3.3.2.31 with an in-house library. The results raw data is valuable in **Supplementary Table 1**. Metabolite levels in THC- or CBD-treated samples were calculated as a percentage of the corresponding metabolite levels in untreated cells from the same mouse.

### 2.4 Blood count analysis

Blood was collected from the tail vein into ethylenediaminetetraacetic acid (EDTA)-coated capillary tubes. Complete blood counts (CBCs) with differentials were performed using a validated BC-2800Vet Auto Hematology Analyzer (Mindray, Shenzhen, China).

### 2.5 Statistical analysis

Data are presented as mean ± standard error (SE). Means were calculated from the indicated number of experiments, with triplicates from each experiment used for analysis. Statistical significance was determined using Kruskal-Wallis test calculator (https://www.statskingdom.com/kruskal-wallis-calculator.html).

### 2.6 Language editing

ChatGPT was used for language editing purposes only.

## 3 Results

### 3.1 Both THC and CBD inhibit activation-induced CD69 and PDL1 expression on B lymphocytes in a dose-dependent manner

**Figure 1:**
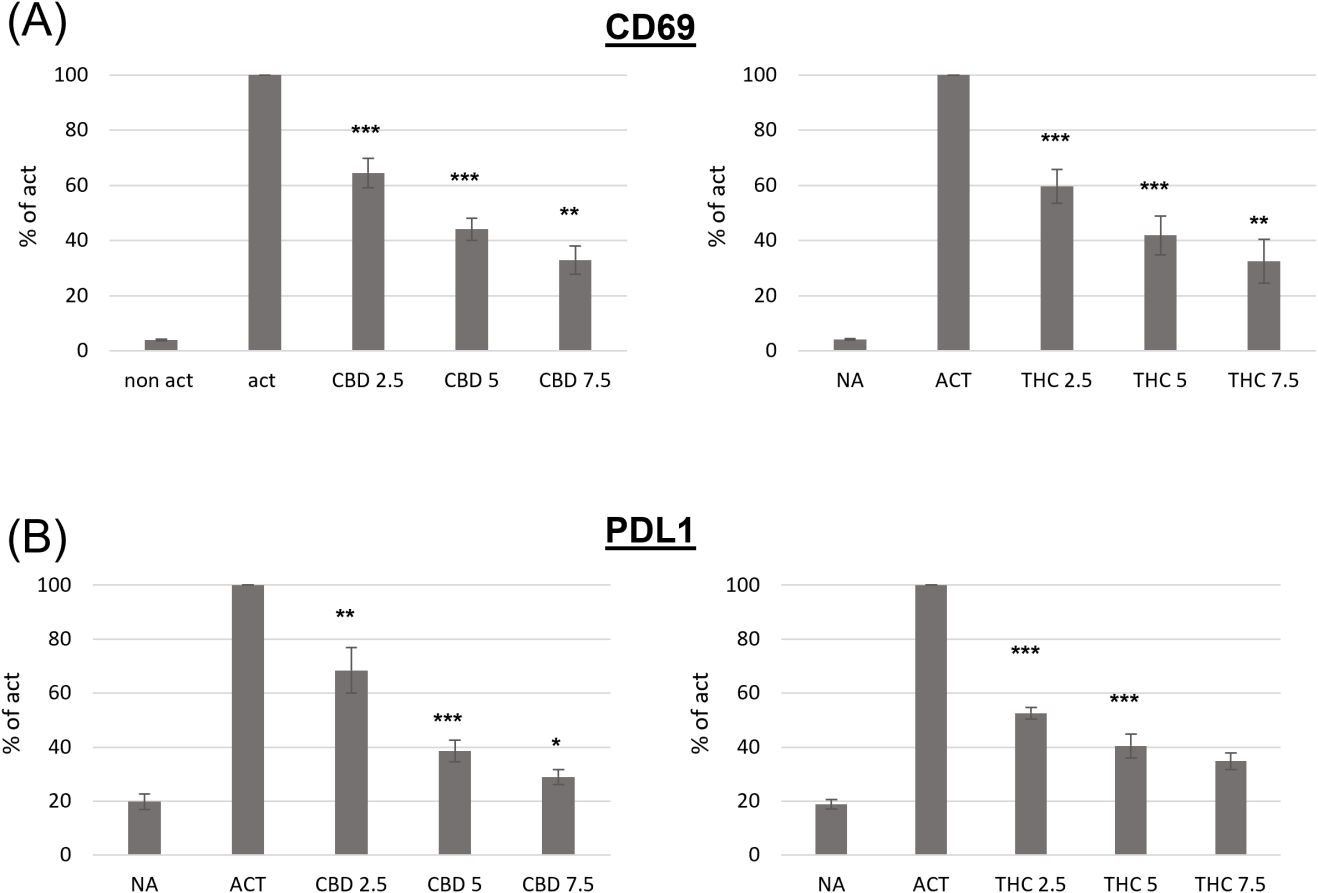
The effect of CBD and THC on the phenotype of activated spleen derived B-cells. Splenocytes were obtained from C57bl/6 female mice and activated for 24h with LPS+IL4, in the presence of cannabinoid treatments. Cell surface expression levels of CD69 (**A**, n=6 per group) and PDL1 (**B**, n=6 per group) on B220 positive cells were determined by flow cytometry. For each mouse, the results are calculated as % of activated sample’s GM. Results expressed as mean +SEM. p value as compare to activated control cells *<0.05; **<0.01; ***<0.001. NA, non-activated; ACT, activated cells; NA, non-activated cells; THC, D9 tetrahydrocannabinol; CBD, cannabidiol.

CD69 is a well-known early leukocyte activation marker. This receptor is rapidly induced in B lymphocytes in response to different activation stimuli, such as lipopolysaccharide (LPS), and plays a role in regulating the B-cell response (18). In order to examine the effect of cannabinoid treatments on B cell activation, we first tested CD69 expression on activated B cells. C57BL/6 mouse splenocytes, or isolated B-cells, were activated *ex vivo* with LPS and IL4 for 24 hours in the presence of THC or CBD in the indicated concentrations. B220 (CD45R), an isoform of CD45, was used as a B-cell marker. CD69 expression on the surface of B220-positive cells was assessed by flow cytometry. Expression levels, relative to activated non-treated cells, were calculated from the geometric mean (GM) values. Our results indicate that both cannabinoids significantly inhibit CD69 expression, in a dose dependent manner (**Figure 1A**, **Supplementary Figure S1A**). For example, both cannabinoids at 2.5 μg/ml inhibited CD69 expression by 35-40%.

Programmed death 1 (PD1) and its ligands, PDL1 and PDL2, deliver inhibitory signals that regulate immune cells, promoting a return to homeostasis after activation. However, elevated PD1/PDL1 expression has also been observed in chronic inflammation and on immune cells from aged mice. We next examined PDL1 expression on the surface of activated B cells in the presence or absence of cannabinoids. We found that both cannabinoids significantly inhibit activation-induced PDL1 expression in a dose-dependent manner (**Figure 1C** **).** For example 2.5 µg/ml of CBD inhibited PDL1 expression by 32%, and 2.5 µg/ml of THC inhibited its expression by 47%.

The inhibitory effects of CBD and THC on the expression of cell-surface molecule was not a result of treatment’s cytotoxicity, since they had no significant effect on cell viability (**Supplementary Figures S2A, B**).

### 3.2 Cannabinoid treatments inhibit activation-induced TNFα secretion, cell proliferation, and IgG secretion in B lymphocytes

The functional consequences of B-cell activation include cytokine secretion, cell proliferation and antibody secretion.

**Figure 2:**
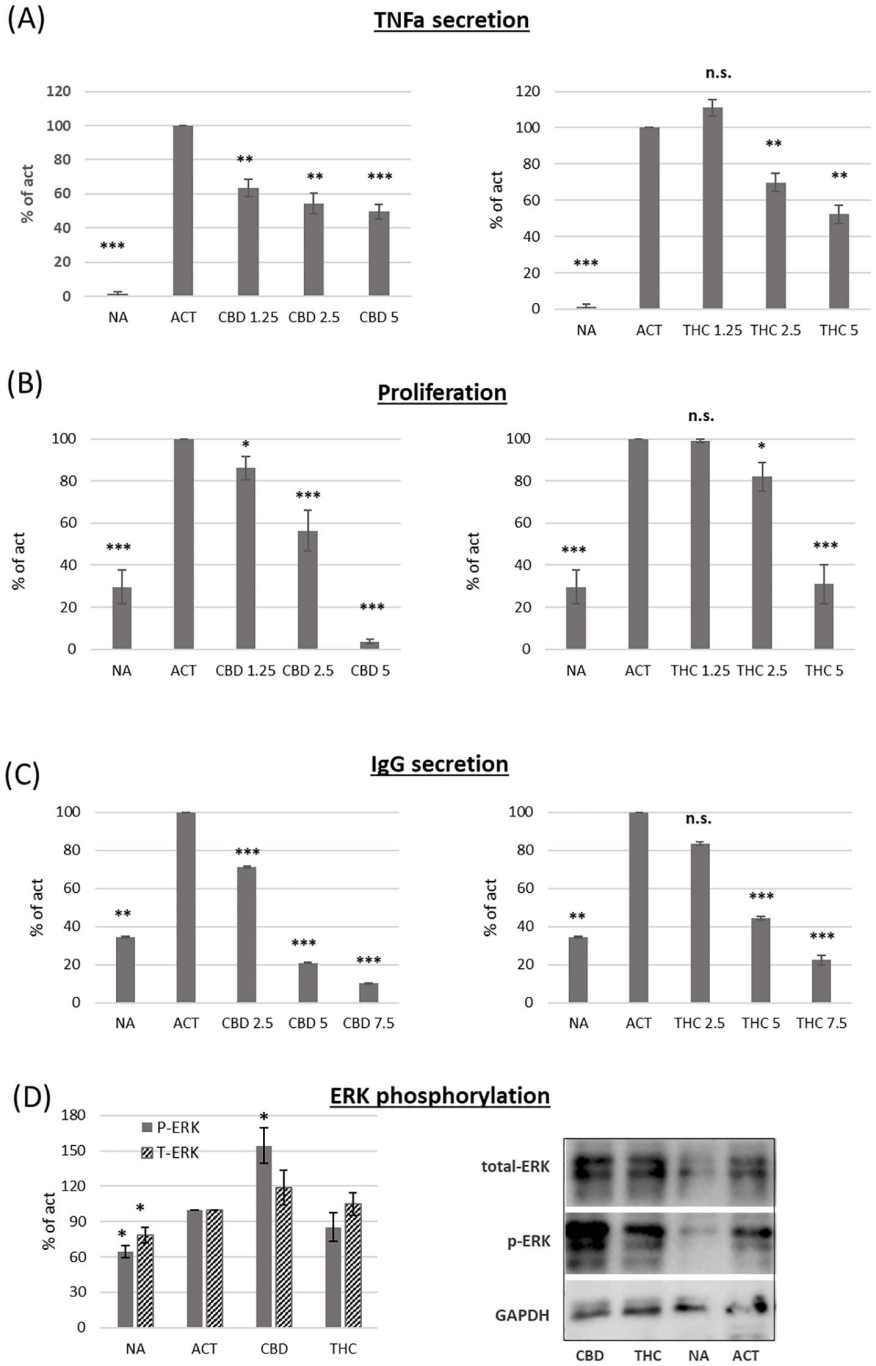
The effect of CBD and THC on activated B-cell function. Isolate B-cells were activated with LPS+IL4, in the presence of cannabinoid treatments (5µg/ml). **(A)** TNF alpha levels in the culture medium after 24h activation, n=6 per group. **(B)** Proliferation of CFSE-stained cells after 96h activation, n=6. **(C)** IgG levels in the culture medium after 72h activation, n=6 per group. **(D)** ERK, p-ERK and GAPDH levels were detected by western blot analysis of Isolated B-cells after 4h incubation with IL4 followed by 30 minutes activation with LPS, n=4 per group. Results are expressed as mean +SEM. p value as compare to activated control cells *<0.05; **<0.01; ***<0.001. NA, non-activated; ACT, activated cells; NA, non-activated cells; THC, D9 tetrahydrocannabinol; CBD, cannabidiol; TNFa, Tumor necrosis factor alpha; IgG, Immunoglobulin G; pERK, phosphorylated ERK; T-ERK, total ERK. n.s., not significant.

We used magnetic beads to isolate B cells via negative selection. The cells were then activated for 24 hours with LPS and IL-4, and TNFα (TNFa) levels in the culture medium were measured using an ELISA assay. Both cannabinoids induced dose-dependent inhibition of TNFα levels (**Figure 2A** **).** However, CBD treatment had a stronger effect. For example, a low concentration of CBD (1.25 µg/ml) inhibited cytokine levels by an average of 27% compared to control samples, whereas the same concentration of THC had no significant effect. To test the influence of cannabinoid treatments on activation-induced B-cell proliferation, we used the carboxyfluorescein succinimidyl ester (CFSE) assay. CFSE-labeled C57BL/6 mouse splenocytes were activated with LPS and IL-4 for 96 hours in the presence of THC or CBD at various concentrations. B-cell proliferation was assessed using CFSE flow cytometry analysis of B220-positive cells. Our results demonstrate dose-dependent inhibition of B-cell proliferation (**Figure 2B** **).** CBD treatment also had a stronger inhibitory effect on B-cell proliferation. For example, 2.5 µg/ml of CBD reduced B-cell proliferation by an average of 44% compared to control samples, whereas 2.5 µg/ml of THC only reduced proliferation by 18%.

IgG levels in the culture medium after 72 hours of activation were measured by ELISA. Our results demonstrate that both cannabinoids reduce IgG levels in a dose-dependent manner (**Figure 2C** **).** However, CBD again had a stronger effect, inhibiting IgG levels by an average of 80% at 5 µg/ml, compared to 56% inhibition in THC-treated samples at the same concentration.

To better understand the inhibitory effect of cannabinoids on B-cell activation, we tested whether cannabinoid treatments affect ERK phosphorylation. Isolated B cells were incubated for 4 hours with IL-4 and then activated for 30 minutes with LPS in the presence of 2.5 µg/ml THC or CBD. ERK, phosphorylated ERK (p-ERK), and GAPDH were detected by western blot analysis. The relative expression levels of ERK and p-ERK were calculated relative to activated controls. Both ERK and p-ERK expression increased upon activation. CBD, but not THC, further increased ERK phosphorylation by an average of 54% compared to control-activated samples (**Figure 2D**).

### 3.3 Decreased lymphocyte counts and elevated PD1/PDL1 expression in the blood of aged mice

**Figure 3:**
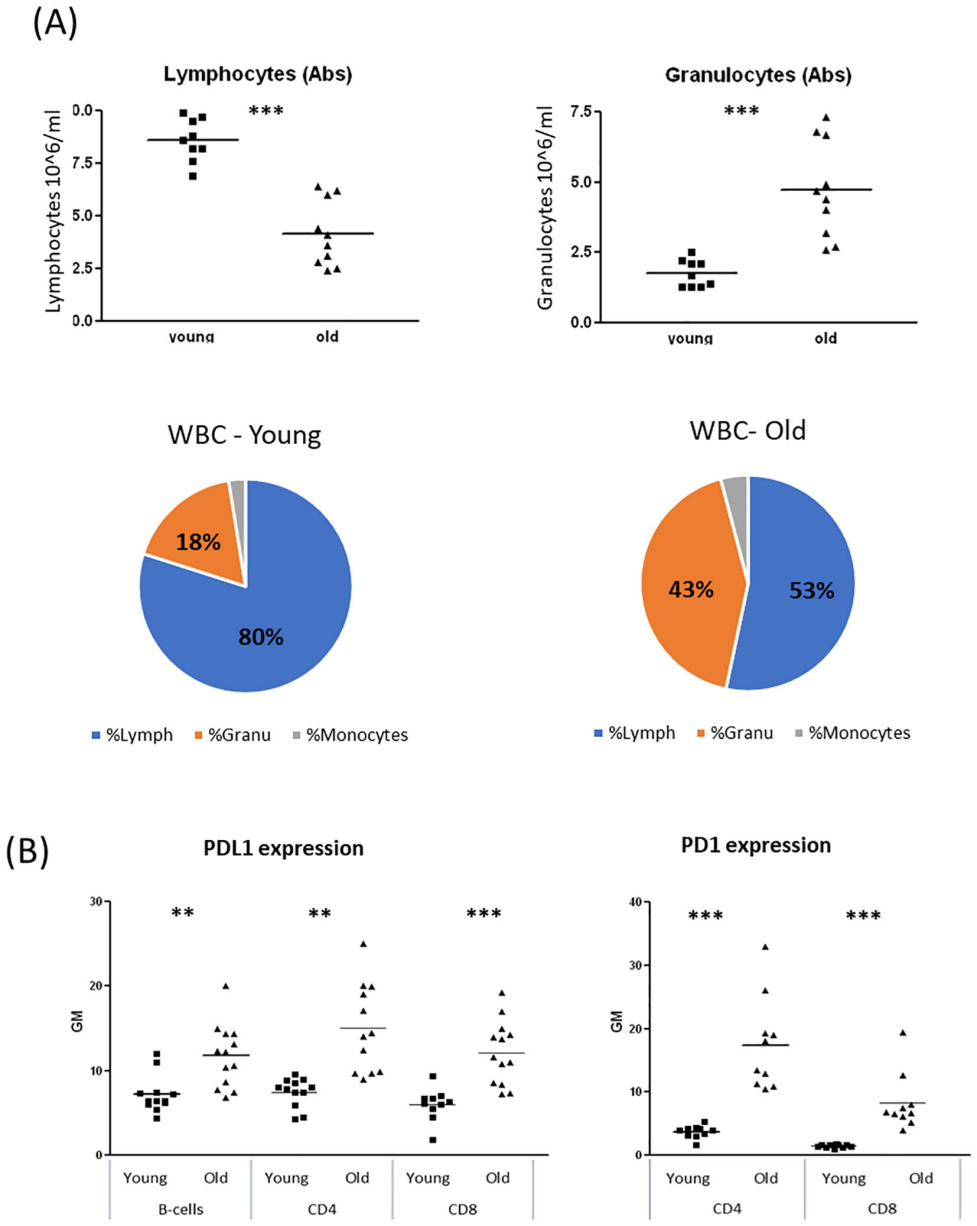
Alteration of WBC populations due to reduced lymphocyte counts and elevated granulocyte counts, in old female mice. Blood samples were collected from young (2–3 month old) and aged (18–20 month old) C57BL/6 mice. **(A)** Complete blood counts (CBC). **(B)** Cell surface expression levels of PDL1 (n=12 per group) and PD1 (n=6 per group) on B-Cells (B220+), CD4 or CD8 positive cells were determined by flow cytometry. Results are expressed as mean +SEM. p value young as compare to old cells **<0.01; ***<0.001.

Evidence suggests that the ECS is influenced by the aging process (16, 17). Age-related changes in the ECS may impair its ability to regulate immune function. Therefore, we sought to investigate whether age-related differences affect B-cell responses to cannabinoid therapies. First, we wanted to evaluate the immune status of the aged mice. To assess hematopoietic changes in the mice, we performed complete blood counts (CBC) on blood samples from young (2–3 months old) and aged (18–20 months old) C57BL/6 mice. We observed alterations in white blood cell (WBC) subpopulations in aged mice, characterized by a reduction in lymphocyte counts and an increase in granulocyte counts (**Figure 3A** **).**

PD1/PDL1 upregulation is recognized as a marker of aging and cellular senescence (19, 20). We examined PD1 expression on CD4^+^ and CD8^+^ T lymphocytes and PDL1 expression on T and B lymphocytes in blood samples from young and aged mice. Both PD1 and PDL1 expression were significantly elevated in lymphocytes from aged mice (**Figure 3B**). Specifically, PD1 expression on aged lymphocytes was 4–5 times higher than in young lymphocytes, while PDL1 expression was 60–90% higher in aged lymphocytes compared to their younger counterparts. Similar trends were observed in spleen lymphocytes (**Supplementary Figure S3**).

### 3.4 B lymphocytes from aged mice respond to cannabinoid treatments

**Figure 4:**
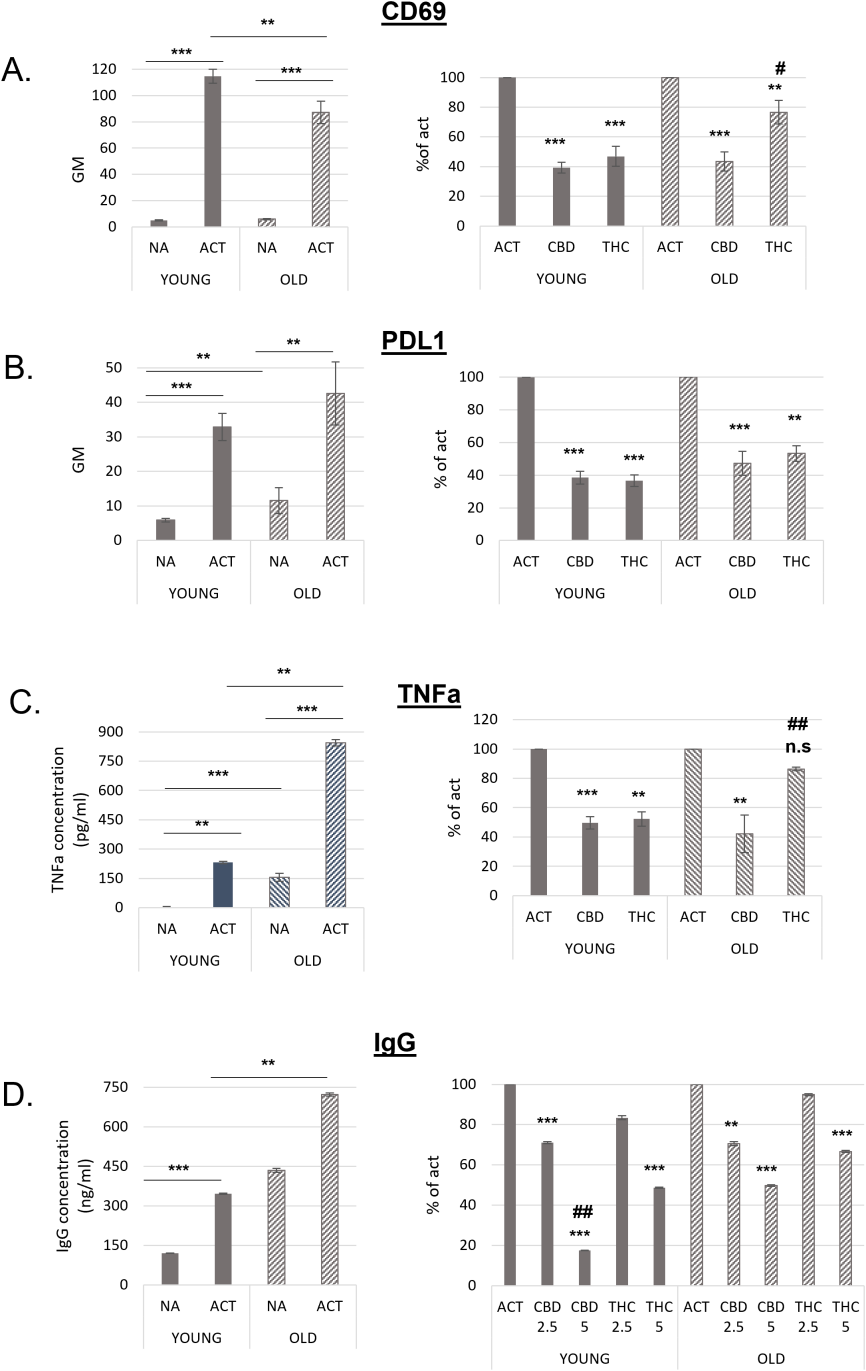
B lymphocytes from old mice are responsive to cannabinoid treatments. Splenocytes were obtained from young and aged C57BL/6 mice. For A and B cells were activated for 24h with LPS+IL4, in the presence of cannabinoid treatments (5µg/ml). Cell surface expression levels of CD69 (**A**, n=12 per group) and PDL1 (**B**, n=8 per group) on B220 positive cells were determined by flow cytometry. **C,** Isolated B-cells were activated with LPS+IL4 for 24h, in the presence of cannabinoid treatments. TNF alpha levels in the culture medium were detected by ELISA, n=6 per group. **D.** splenocytes were activated for 72h. IgG levels in the culture medium were measured by ELISA, n=7 per group. Results are expressed as mean +SEM. Results in the right side of the figure are calculated as % of activated sample’s GM, for each mouse. Results expressed as mean +SEM. Relative p value as compare to activated control cells **<0.01; ***<0.001. p value THC compare to CBD treated cells #,<0.05; ##,<0.01; NA-non-activated ACT–activated cells, NA, non-activated cells; THC, D9 tetrahydrocannabinol; CBD, cannabidiol; TNFa, Tumor necrosis factor alpha; IgG, Immunoglobulin G. n.s., not significant.

Next, we compared the responsiveness of activated B cells from young and aged mice to THC and CBD treatments. Splenocytes from both groups were activated with LPS and IL-4 for 24 hours in the presence of 5 µg/mL THC or CBD. Following treatment, CD69 (**Figure 4A**) and PDL1 (**Figure 4B**) expression on B220^+^ cells was assessed by flow cytometry. As shown in **Figure 4A** (left), activated B cells from aged mice exhibited significantly lower CD69 expression compared to those from young mice. When compared to their respective activated controls, CBD treatment inhibited CD69 expression to a similar extent in both young and aged cells; However, THC treatment reduced CD69 expression by only 23% in aged samples, significantly less than the 53% inhibition observed in young samples (**Figure 4A**, right**).**

Non-activated (rested) cells from aged mice expressed higher levels of PDL1. However, PDL1 expression increased upon activation in both groups (**Figure 4B**, left). The inhibitory effect of cannabinoids on PDL1 expression was slightly lower in aged B cells. This difference reached statistical significance only in the THC-treated samples, where inhibition was 47% in aged cells compared to 63% in young cells (**Figure 4B**, right).

The inhibitory effects of CBD and THC on the expression of CD69 and PDL1 was not a result of treatment’s cytotoxicity, since they had no significant effect on cell viability (**Supplementary Figure S2C**).

TNFα levels in the culture medium of isolated B cells also increased upon activation in both groups. However, significantly higher TNFα levels were observed in both non-activated and activated samples from aged mice compared to those from young mice (**Figure 4C**, left). CBD efficiently reduced TNFα levels by 50–60% in both age groups, whereas THC significantly decreased TNFα levels only in young samples (**Figure 4C**, right).

Recent studies have shown that plasma IgG levels increase with age in mice, leading to IgG accumulation in tissues, fibrosis, and metabolic decline (21). To compare the inhibitory effects of cannabinoids on IgG secretion, we activated splenocytes from young and aged mice for 72 hours and measured IgG levels in the culture medium by ELISA. IgG levels in both non-activated and activated aged cells were significantly higher than in young cells (**Figure 4D**, right). Notably, non-activated aged cells secreted more antibodies than activated young cells, and IgG secretion further increased upon activation. Cannabinoid treatments at higher concentrations were less effective at inhibiting IgG secretion in aged cells (**Figure 4C**, left). Specifically, 5 µg/mL CBD reduced IgG levels by an average of 83% in young samples but only by 50% in aged samples compared to their respective activated controls. Similarly, 5 µg/mL THC reduced IgG levels by 51% in young samples but only by 33% in aged samples.

### 3.5 THC- and CBD-treated activated B lymphocytes exhibit distinct metabolite profiles

**Figure 5:**
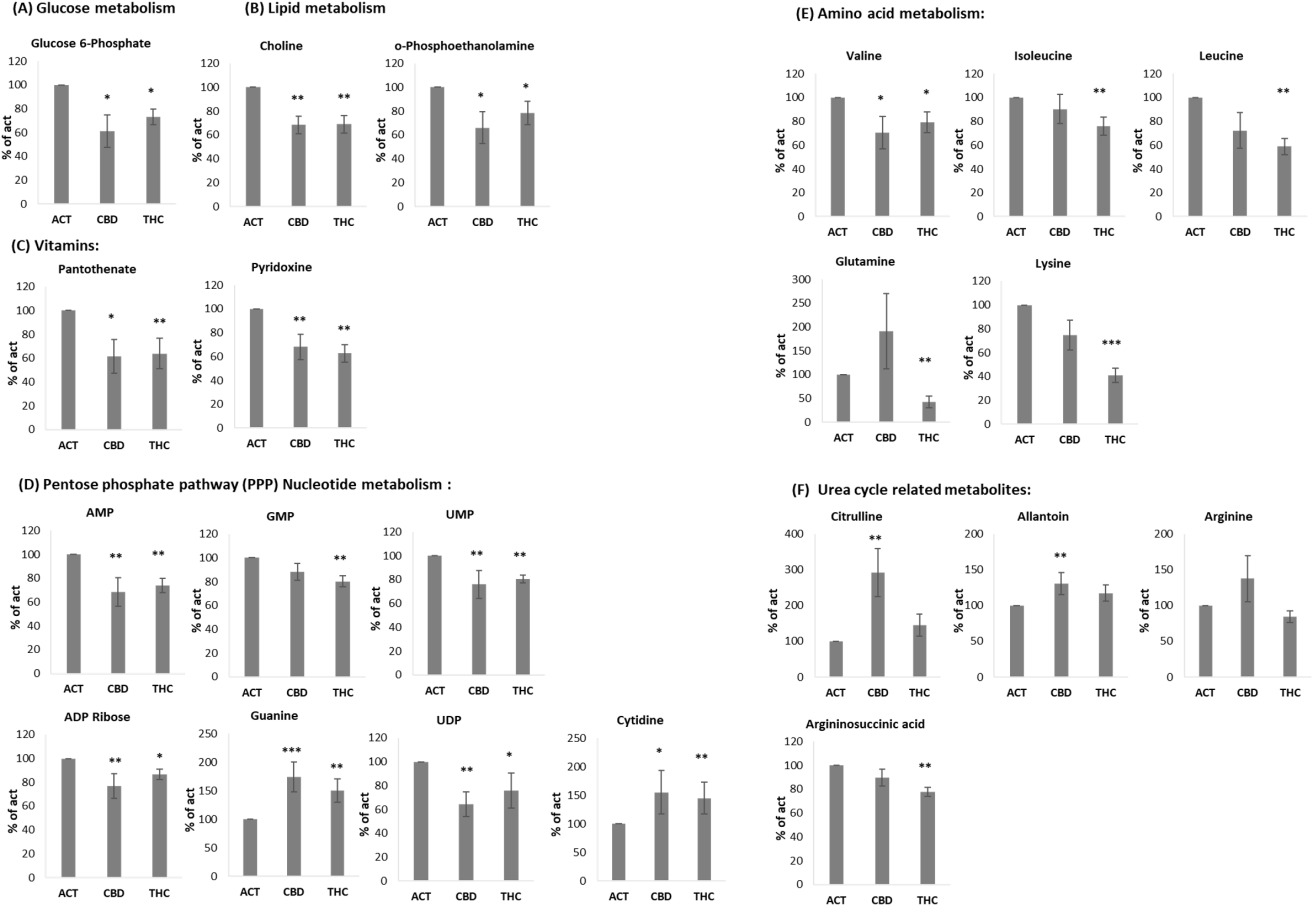
THC and CBD treated activated B lymphocytes show different metabolite composition. LCMS metabolomics analysis of isolated B-cells, activated with LPS+IL4, in the presence of cannabinoid (5µg/ml), n=7 per group, including 4 young and 3 aged mice. Mean of each treatment is marked. p value as compare to activated control cells *<0.05; **<0.01; ***<0.001. ACT, activated cells; THC-D9, tetrahydrocannabinol; CBD, cannabidiol.

Accumulating evidence suggests that THC and CBD exert their effects on mammalian cells through different set of receptors (3). Additionally, these two cannabinoids have been shown to produce different effects in the nervous system (2). However, our findings, along with previous studies, indicate that both THC and CBD inhibit immune cell activation and effectively reduce inflammatory responses. Based on this, we hypothesized that cannabinoid treatments may modulate immune activation by altering cellular metabolism. To test this hypothesis, isolated B cells from young (n = 4) and aged mice (n = 3) were activated for 24 hours in the presence of 5 µg/mL THC or CBD. Cell pellets were then subjected to LC-MS metabolomics analysis. Metabolite levels in THC- or CBD-treated samples were calculated as a percentage of the corresponding metabolite levels in untreated cells from the same mouse. **Figure 5** summarizes metabolites that were significantly increased or decreased in at least one of the treatment groups. Since the metabolic profiles of young and aged mice were largely similar, data from both groups were combined in the analysis. Raw data of the LC-MS metabolomics is attached as **Supplementary Table 1**.

Several metabolites were significantly reduced by both THC and CBD. These included glucose-6-phosphate (**Figure 5A**), as well as choline and O-phosphoethanolamine, which are involved in lipid metabolism (**Figure 5B**). In addition, the vitamins pantothenate (vitamin B5) and pyridoxine (vitamin B6) were similarly reduced by both cannabinoids (**Figure 5C**). A comparable effect was observed in metabolites involved in the pentose phosphate pathway (PPP) and nucleotide metabolism, including AMP, GMP, UMP, ADP ribose, guanine, UDP, and cytidine (**Figure 5D**), as well as the amino acid valine (**Figure 5E**). However, THC selectively reduced a distinct set of amino acids, including isoleucine, leucine, glutamine, and lysine—the latter showing an average reduction of 59% (**Figure 5E**). Interestingly, some of the most significant results are related to Urea cycle metabolites. CBD treatment increased the levels of Citrulline and Allantoin, compared to activated control, in addition to a trend of elevation in Arginine, which did not reach significance (**Figure 5F**). THC treatment reduced the levels of Arginosuccinic acid (**Figure 5F**).

## 4 Discussion

B lymphocytes play key roles in immunity but also contribute to the pathogenesis of various diseases (22). The endocannabinoid system plays a crucial role in mature B-cell function, as demonstrated in multiple studies. Eisenstein et al. reported that anandamide induces dose-dependent immunosuppression in plaque-forming cell assays of antibody formation (23) Similarly, Sido et al. used an *in vivo* delayed-type hypersensitivity model to show that 2-AG production by activated B and T cells modulates inflammation (24). Notably, Dotsey et al. demonstrated that CB2 ligation reduces vaccination-induced immunity, and transient administration of CB2 antagonists during immunization enhanced the intensity and breadth of antigen-specific antibody responses in both young and aged mice (11). The effects of several phytocannabinoids on B-cell immunity have also been documented. For instance, exposure of peripheral blood mononuclear cells to a cannabinoid mixture significantly alters cytokine profiles and induces caspase-dependent apoptosis (25). Additionally, THC has been shown to suppress IgM release from activated B lymphocytes by impairing plasmacytic differentiation (26). The immune system undergoes numerous age-related alterations that result in a clinically significant decline in function, increasing morbidity and mortality from infectious and chronic diseases. Changes in endocannabinoid concentrations and ECS dysregulation have also been linked to aging. For example, naturally aged mice exhibit a marked decrease in 2-arachidonoylglycerol (2-AG) levels and increased activity of MAGL, its primary degrading enzyme, in the hippocampus (27).

In this study, we investigated the effects of THC and CBD on primary B cells from both young and aged mice, including their impact on activation, humoral function, and, for the first time, cell metabolism. We used several well-established surface marker proteins to characterize B-cell activation under cannabinoid treatment, specifically CD69, and PD1/PDL1.

CD69, an early lymphocyte activation marker, regulates lymphocyte migration dynamics in the spleen (28), and therefore was used a marker for activated B cells in our experiments. The dose-dependent decrease in CD69 expression suggests a direct suppressive effect of cannabinoids on B-cell activation. Moreover, while CBD similarly affected CD69 expression in both young and aged B cells, THC was significantly less potent in reducing CD69 expression in primary B cells from aged animals compared to young ones.

Early B-cell activation is primarily driven by IL-4 via the PARP14-STAT6 pathway, which does not involve ERK1/2, whereas BCR/TLR4-LPS signaling requires ERK1/2 phosphorylation (29–32). Our data showed that CBD treatment enhanced ERK1/2 phosphorylation, potentially disrupting this pathway and leading to suppressed activation and CD69 expression while sparing IL-4 signaling. In contrast, THC inhibited CD69 expression without affecting ERK1/2 phosphorylation, indicating a distinct mechanism of action. These findings align with prior studies showing that cannabinoids mediate their effects through different receptors (3).

The PD1/PDL1 pathway is essential for immune homeostasis post-activation and serves as an immune checkpoint. The PD1 receptor modulates T-cell activity, while its ligand, PDL1 can be found on various immune cells (33), including B cells, and its expression is up-regulated upon stimulation (34, 35). Our results demonstrate that cannabinoids significantly suppress PDL1 expression on activated B cells. PDL1 plays a key role in B-cell function and immune regulation. It influences humoral immune responses, conditionates the selection and survival of late germinal center (GC) B cells, and participates in the function of B regulatory cells (34). PD1/PDL1 pathway is also one of the pathways that are targeted for immunotherapy, since this pathway is involved in the induction and maintenance of immune tolerance within the tumor microenvironment. Cancer patients are often treated with cannabis-based treatments for symptoms management, therefore; the effect of cannabinoids on PDL1 expression in B cells and other cancerous and non-cancerous cells should be taken under consideration in treatment management. Indeed, Cannabis consumption used by cancer patients during Immunotherapy was correlates with poor clinical outcome (36).

Aging is concurrent with a slow and constant functional deterioration of the immune system, known as immunosenescence, which is accompanied by an increase in chronic inflammatory processes, a phenomenon known as “Inflammaging” (37). We observed elevated PD1/PDL1 expression on lymphocytes from aged mice, which was further increased upon activation but suppressed by cannabinoid treatments (38). B-cell percentages and numbers decline significantly with age in humans (39–43), and similar age-related immune alterations were evident in our murine model. Immunosenescence was demonstrated by a reduced CD69 response upon activation. Although TNFα levels were elevated in aged B cells, the fold increase upon activation was significantly lower than in young B cells. Inflammaging was indicated by increased PD1/PDL1 expression, TNFα secretion, and robust IgG production from non-activated B cells. Notably, IgG secretion from activated B cells in aged mice was less susceptible to phytocannabinoid suppression compared to young mice. Additionally, CD69 expression and TNFα secretion were less affected by THC in aged B cells, potentially reflecting age-related alterations in the ECS. Such alterations were previously demonstrated mainly in the brain (17, 27, 44).

We also examined the impact of cannabinoids on B-cell metabolism. Glucose metabolism is central to B-cell activation, supplying energy and biosynthetic precursors (45, 46). Both THC and CBD significantly reduced glucose-6-phosphate levels, as well as key metabolites involved in lipid metabolism and the pentose phosphate pathway (PPP) (47). Notably, choline, a precursor for monoacylglycerol, was also reduced, which could impact ECS regulation (48). Furthermore, both cannabinoids lowered levels of vitamins B5 (pantothenate) and B6 (pyridoxine), which are critical for energy metabolism and immune function (49, 50).

THC treatment specifically, reduced the level of several amino acids, including glutamine, Isoleucine, Leucine, Valine and Lysine. Although amino acids are well known as substrates for protein synthesis, they are also metabolized as energy sources and as substrates for functional catabolites and therefore have important contribution to immune cell activation (51). Glutamine metabolism, for example, is important for the proliferation, differentiation and effector functions of immune cells by generating adenosine triphosphate (ATP) and biosynthetic precursors through the tricarboxylic acid (TCA) cycle. The branched-chain amino acids, leucine, isoleucine and valine, regulate T-cell activation via the mTOR pathway (52). Additionally, in muscle cells, Leucine and isoleucine increase translocation of the glucose transporters GLUT1 and GLUT4 to the cell surface (53, 54), affecting B cell differentiation. Lysine, a precursor for L-carnitine, plays a role in mitochondrial fatty acid oxidation and immune regulation (55).

In contrast, CBD had minimal effects on these amino acids but significantly increased citrulline levels, suggesting a shift towards urea cycle metabolism. This finding aligns with emerging research on citrulline’s role in immune regulation, including its involvement in memory T-cell formation (56) and suppression of pro-inflammatory signaling in macrophages signals (57). CBD also elevated allantoin levels, a metabolite with anti-inflammatory properties (58–60), while THC reduced argininosuccinate without significantly altering citrulline or allantoin levels. Overall, our results highlight distinct metabolic effects of THC and CBD on B-cell activation.

## 5 Conclusions

Although both cannabinoids suppressed B-cell activation, we show here, for the first time, differences in their mechanisms of action. CBD influenced signal transduction by inducing ERK phosphorylation and directing metabolism towards the urea cycle, while THC primarily reduced amino acid availability. Additionally, their effects varied between young and aged B cells, suggesting that age-related ECS changes may influence cannabinoid sensitivity. These findings emphasize the need for further investigation into phytocannabinoid-based therapies, particularly for age-specific applications. Given the immunoregulatory properties of cannabinoids, especially CBD, tailored therapeutic strategies may be developed to optimize their clinical use.

## Data Availability

The original contributions presented in the study are included in the article/**Supplementary Material**, further inquiries can be directed to the corresponding author/s.

## Glossary

2-AG: 2-Arachidonoylglycerol
ATP: Adenosine triphosphate
AMP: Adenosine monophosphate
GMP: Guanosine monophosphate
BCR: B cell receptor
CB2: Cannabinoid receptor 2
CBC: Complete blood count
CBD: Cannabidiol
CD: Cluster of differentiation
CFSE: Carboxyfluorescein succinimidyl ester
DCM: Dichloromethane
ECL: Enhanced chemiluminescence
ECS: Endocannabinoid system
EDTA: Ethylenediaminetetraacetic acid
ELISA: Enzyme-linked immunosorbent assay
ERK: Extracellular signal-regulated kinase
FCS: Fetal calf serum
GC: Germinal center
GLUT: Glucose transporter
GM: Geometric mean
IgG: Immunoglobulin G
IL: Interleukine
IL-4: Interleukin-4
LC-MS: Liquid chromatography–mass spectrometry
LPS: Lipopolysaccharide
LPS: Lipopolysaccharide
MAGL: Monoacylglycerol lipase
mTOR: mammalian target of rapamycin
PARP14: Poly(ADP-ribose) polymerase family member 14
PD1: programmed death 1
PDL1: programmed death 1 ligand
p-ERK: Phosphorylated extracellular signal-regulated kinase
PPP: Pentose phosphate pathway
SDS-PAGE: Sodium dodecyl sulfate polyacrylamide gel electrophoresis
SE: Standard error
STAT6: Signal transducer and activator of transcription 6
TCA: Tricarboxylic acid
THC: Δ^9^-Tetrahydrocannabinol
TLR4: Toll-like receptor 4
TNFα: Tumor necrosis factor α
UDP: Uridine diphosphate
WBC: White blood cell
