# Supplementary material for: Differential metabolic pathways underlie THC- and CBD-mediated inhibition of B-cell activation in both young and aged mice
Source: Front Immunol. 2025 Jun 17;16:1605474. doi: 10.3389/fimmu.2025.1605474 (PMC12208840; doi:10.3389/fimmu.2025.1605474)
Supplement: Supplementary file 1 [file DataSheet1.pdf]

Supplementary material:

Supplementary figure 1. examples for FACS analysis (non activated and activated splenocytes)

NA ACT

A. **Duplicates determined. No gate**

B. **Gate 1 determined. Dup gate**

C. **B220 positive cells determined. Gate 1**

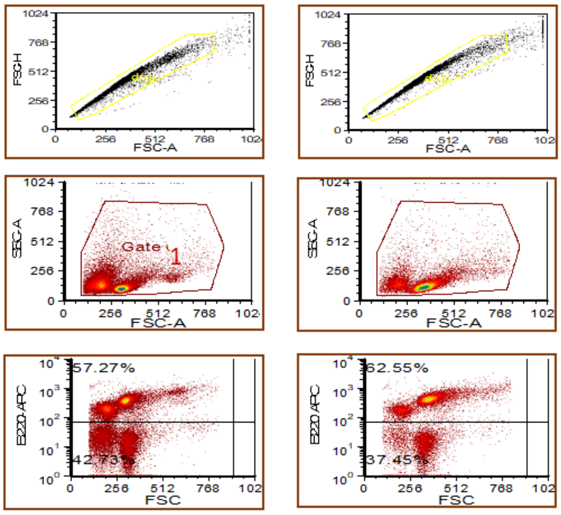

D. **CD69 expression on B220 positive cells**

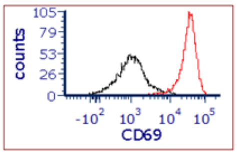

	Median	Arithmetic mean
NA	1030	1609
ACT	33524	35019

E. **PDL1 expression on B220 positive cells**

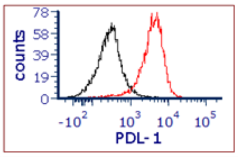

	Median	Arithmetic mean
NA	328	458
ACT	3652	4127

F. **B220/CD3 and B220/CD11b double staining (activated cells)**

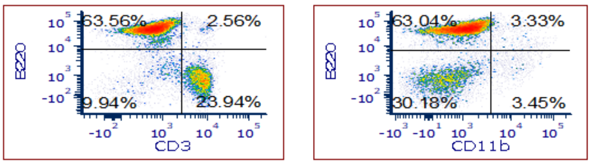

Splenocytes were obtained from C57bl/6 female mice and activated for 24h with LPS+IL4, in the presence of cannabinoid treatments. Non-adherent cells were collected and cell-surface expression levels of CD69 and PDL1 on B220 positive cells were determined by flow cytometry.

Supplementary figure 2. The effect of cannabinoid treatments on cell viability

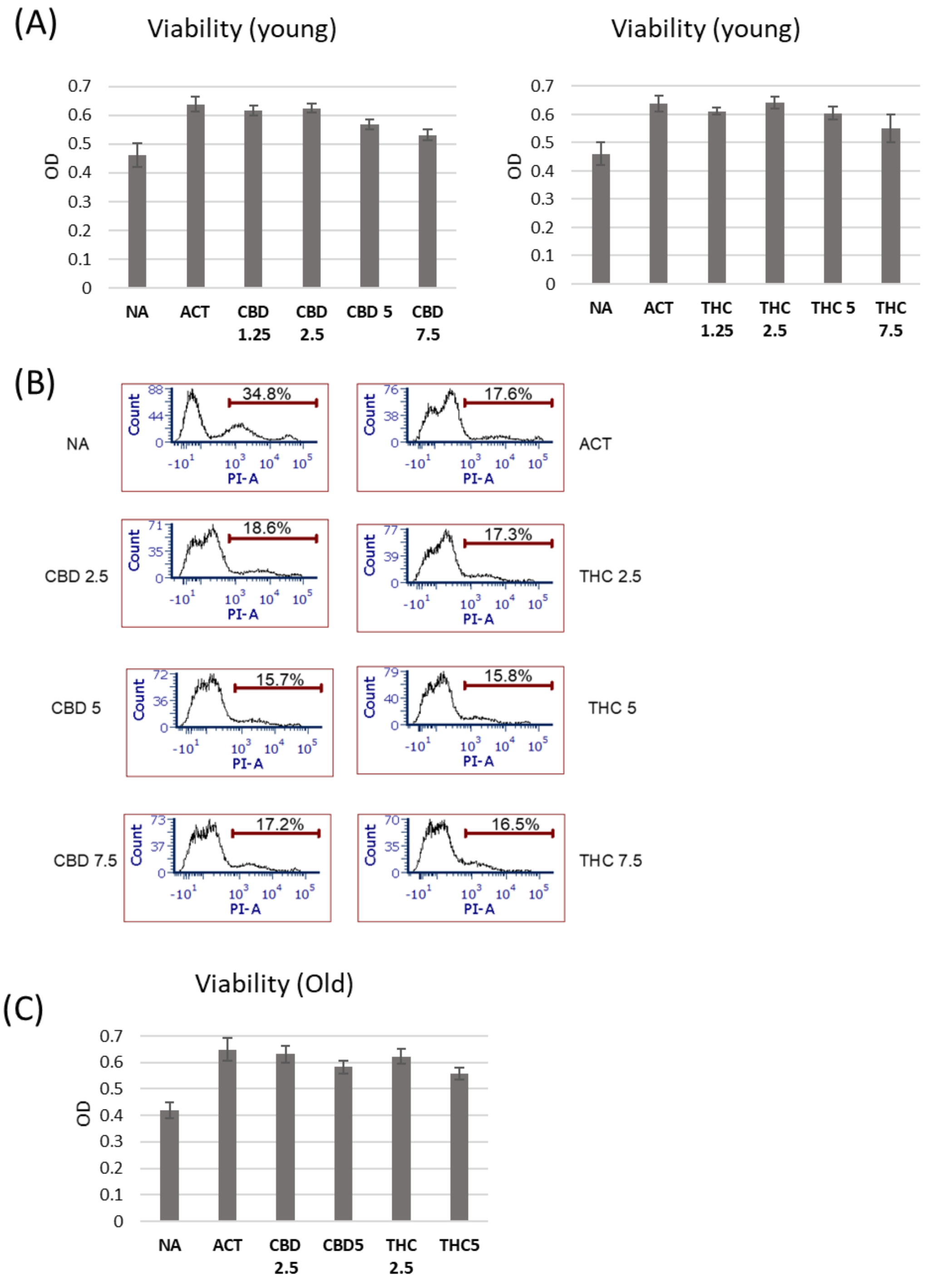

Splenocytes were obtained from young (A,B) and aged (C) C57BL/6 mice. Cells were activated for 24h with LPS+IL4, in the presence of cannabinoid treatments. (A,C) Cell viability was examined using XTT based colourimetric assay (Biological Industries, Israel). (n=4 per group). (B) Cell viability was examined using PI staining in Flow cytometry analysis.

Supplementary figure 3. PDL1/PD1 on spleen lymphocytes

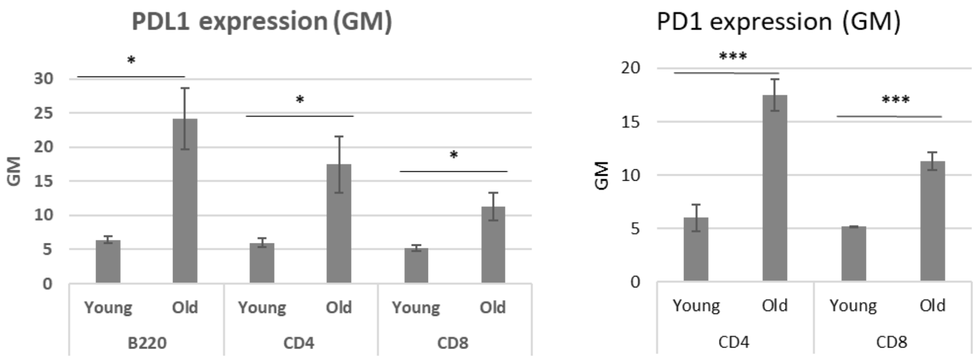

Splenocytes were extracted from young and aged C57BL/6 mice. Cell surface expression levels of PDL1 (n=3 per group) and PD1 (n=3 per group) on B220, CD4 or CD8 positive cells were determined by flow cytometry.
